# Supplementary material for: Examining Gender Differences and Their Associations Among Psychosocial Distress, Social Support, and Financial Well-Being of Informal Caregivers of Older Adults in the Rural Northcentral United States
Source: Healthcare (Basel). 2025 Dec 20;14(1):17. doi: 10.3390/healthcare14010017 (PMC12785571; doi:10.3390/healthcare14010017)
Supplement: Supplementary file 1 [file healthcare-14-00017-s001.zip › healthcare-4012931-supplementary.pdf]

Supplemental Materials

Supplemental Table S1. The Financial Well-Being Scale, the Oslo-3 Social Support Scale, and the Psychosocial Distress measure

**CFPB Financial Well-Being Scale** [1]					
	Completely	Very well	Somewhat	Very little	Not at all
1. Because of my money situation, I feel like I will never have the things I want in life					
2. I am just getting by financially					
3. I am concerned that the money I have or will save won't last					
	Always	Often	Somewhat	Rarely	Never
4. I have money left over at the end of the month					
5. My finances control my life					
**The Oslo-3 Social Support Scale (OSSS-3)** [2]					
1: How many people are so close to you that you can count on them if you have great personal problems?	None	1-2	3-5	5+	
2: How much interest and concern do people show in what you do?	None	Little	Uncertain	Some	A lot
3: How easy is it to get practical help from neighbors if you should need it?	Very difficult	Difficult	Possible	Easy	Very easy
**Psychosocial Distress measure**					
	Measured on a scale from 0 to 10, with 0 indicating the lowest level of distress and 10 indicating the highest				
1. I DO NOT HAVE THE ORIGINAL QUESTIONAIRE, PLEASE ADD THE FIVE ITMES HERE. stressed					
2. anxious					
3. depressed					
4. lonely					
5. having no time for oneself					

Supplemental Table S2. Conversion of CFPB Financial Well-Being Raw Scores to Standardized Scores [1]

Total Raw Score	Standardized Scores			
	Self-administered		Administered by someone else	
	18–61 years	62+ years	18–61 years	62+ years
0	19	20	22	24
1	25	26	30	30
2	29	31	33	33
3	32	34	36	37
4	36	37	39	39
5	38	40	42	42
6	41	43	44	44
7	43	46	47	46
8	46	48	49	49
9	48	51	51	51
10	50	53	54	53
11	53	55	56	55
12	55	58	58	57
13	57	61	60	59
14	60	63	63	62
15	63	66	66	64
16	65	69	68	67
17	68	73	72	70
18	72	76	75	73
19	76	81	80	77
20	82	90	87	84

References

1. Measuring financial well-being: A guide to using the CFPB Financial Well-Being Scale. https://files.consumerfinance.gov/f/201512_cfpb_financial-well-being-user-guide-scale.pdf

2. Kocalevent, RD., Berg, L., Beutel, M.E. et al. Social support in the general population: standardization of the Oslo social support scale (OSSS-3). *BMC Psychol* **6**, 31 (2018). https://doi.org/10.1186/s40359-018-0249-9
